# Anti-Hu-Associated Encephalomyelitis as a Presentation of Primary Extrapulmonary Small Cell Carcinoma of the Small Bowel: A Case Report

**DOI:** 10.7759/cureus.33605

**Published:** 2023-01-10

**Authors:** Vincent Trung H Ngo, Michael P Meyers, Kasim Qureshi, Muhammad Umar Farooq

**Affiliations:** 1 Neurology, Trinity Health Saint Mary's - Grand Rapids, Grand Rapids, USA; 2 Internal Medicine, Trinity Health Saint Mary's - Grand Rapids, Grand Rapids, USA; 3 Hauenstein Neurosciences Center, Trinity Health Saint Mary's - Grand Rapids, Grand Rapids, USA

**Keywords:** ki-67 expression, small cell carcinoma, paraneoplastic encephalomyelitis, neuroendocrine tumor, case report, anti-hu, neuroendocrine carcinoma, extrapulmonary small cell carcinoma, paraneoplastic syndrome

## Abstract

Small cell carcinoma (SCC) is a neuroendocrine tumor (NET) commonly found in the lung, known for rapid proliferation and early metastasis. Extrapulmonary small cell carcinomas (ESCC) are rare, with GI tract carcinomas exceedingly so. Due to the lack of clinical data on the treatment of ESCC, the standard regimen is the same as the SCC of the lung. Documented accounts of paraneoplastic encephalomyelitis associated with NETs are also uncommon. We present a patient who suffered from neurologic deficits before being diagnosed with paraneoplastic encephalomyelitis from a duodenal ESCC.

The patient presented with ear pain and hematemesis. New symptoms arose after the resolution of initial symptoms, including shortness of breath and numbness. Autoimmune workup was positive for anti-Hu antibodies. A position emission tomography (PET) scan showed increased uptake in the duodenal region. Biopsy results from a duodenal ulcer revealed poorly differentiated neuroendocrine carcinoma with positive synaptophysin and strong positivity of Ki-67, consistent with ESCC. Numerous treatments, including platinum-based chemotherapy, yielded no neurologic improvement for the patient. This case details an atypical presentation of ESCC, which should be considered in patients suspected of paraneoplastic encephalomyelitis.

## Introduction

Anti-Hu-associated encephalomyelitis is a rare entity that presents with various acute to subacute neurologic manifestations. It is a type of paraneoplastic encephalitis and is typically associated with small cell carcinoma (SCC) of the lung, a type of neuroendocrine carcinoma (NEC) known for rapid proliferation and early metastasis [1,2]. Rarely, it is also associated with extrapulmonary small cell carcinoma (ESCC). ESCC is rare, with documented accounts of GI tract SCC being exceedingly so [3]. When paraneoplastic encephalitis is suspected, an exhaustive search for underlying malignancy is necessary. The syndrome may predate the discovery of malignancy by months or years in some cases.

The 2019 WHO classification of tumors of the digestive system classifies poorly differentiated neuroendocrine tumors (NET) as NECs. NECs are high grade with elevated mitotic rate and Ki-67; they are sub-divided into small-cell and large-cell types [4]. ESCCs of the GI tract can present as NETs or NECs, depending on the level of differentiation. For the small bowel, the incidence of both neuroendocrine tumors (NETs), as well as NECs, has been rising [3,5]. Documented cases of NETs and NECs involving paraneoplastic encephalomyelitis have also risen, with a majority resulting from colon cancer rather than carcinomas of the small bowel [6,7]. The rarity of NETs and NECs, as well as the broad neurologic symptoms of paraneoplastic encephalitis, poses a diagnostic challenge as they are often considered later in the disease course after ruling out more common diseases. We present a patient who suffered from various neurologic deficits, including neuromuscular weakness leading to respiratory failure and was eventually diagnosed with paraneoplastic encephalomyelitis due to ESCC of the duodenum. This case highlights both the importance of considering rare neoplasms in patients with paraneoplastic encephalomyelitis and the need to investigate effective treatments. 

## Case presentation

A 53-year-old female presented with right-sided otalgia for three days, along with intermittent paresthesia and tinnitus, after using firearms at a gun range. Gabapentin 100 mg three times daily was started and switched to duloxetine 30 mg daily, which was effective in relieving the pain, but not paresthesia. Two months after the initial onset of otalgia, she presented to the emergency department (ED) multiple times for hematemesis. She was ultimately admitted and started on intravenous (IV) pantoprazole 40 mg twice daily. An upper GI endoscopy showed a cratered ulcer in the duodenum, with a biopsy negative for malignancy.

Four months later, the patient presented to her primary care provider (PCP) with complaints of new onset generalized weakness and paresthesia in her tongue, hands, and dorsum of the right foot. Electromyography (EMG) obtained at this time was suggestive of sensory axonal polyneuropathy in the upper extremities. Two months after the onset of new paresthesia, the patient began to have binocular diplopia along with left-beating nystagmus. The symptoms became distressing to the patient, and thus she was started on bupropion 150 mg daily. Three weeks after, the patient was brought into the ED for generalized tonic-clonic seizures. An electroencephalogram (EEG) was not pursued, as the patient had returned to her baseline. Bupropion was discontinued, and the patient was started on lacosamide 100 mg twice daily.

**Figure 1:**
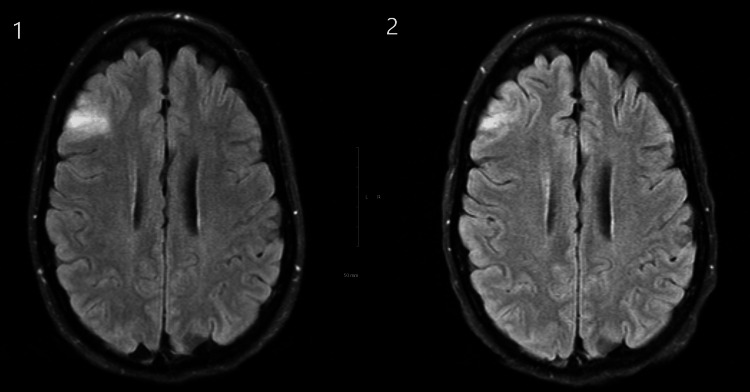
MRI of the brain T2 FLAIR showing hyperintensity in the right lateral frontal lobe during early diagnostic workup (1) and later in the disease course after treatment (2) Treatments included IVIg, plasmapheresis, and rituximab. FLAIR - fluid-attenuated inversion recovery; IVIg - intravenous immunoglobulin

After her discharge from the hospital, the patient was given an extensive outpatient workup for possible paraneoplastic encephalitides. Other diagnoses that were considered included compressive neuropathies, cervical radiculopathies, a systemic inflammatory process, and idiopathic polyneuropathy. The patient did not have any history of cancer, although her father did have skin cancer with liver metastasis. Magnetic resonance imaging (MRI) of the brain was unremarkable except for a small area of hyperintensity in the T2 and fluid-attenuated inversion recovery (FLAIR) in the right lateral frontal lobe (Figure 1). An extensive autoimmune and paraneoplastic panel was positive for anti-GAD-65 and type 1 antineuronal nuclear (ANNA-1) or anti-Hu antibodies. Associated positive labwork was speckled antinuclear antibody (ANA) with titers of 1:80, which was nonspecific although initially suggested the possibility of an autoimmune cause of the patient's symptoms. Of note, her labwork was negative for any myasthenic antibodies, including acetylcholine receptor binding, blocking, and modulating antibodies, as well as striated muscle kinase antibodies. A lumbar puncture showed increased levels of immunoglobulin G (IgG) and was positive for oligoclonal bands. A computed tomography (CT) scan of the chest was negative for small-cell lung carcinoma.

**Figure 2:**
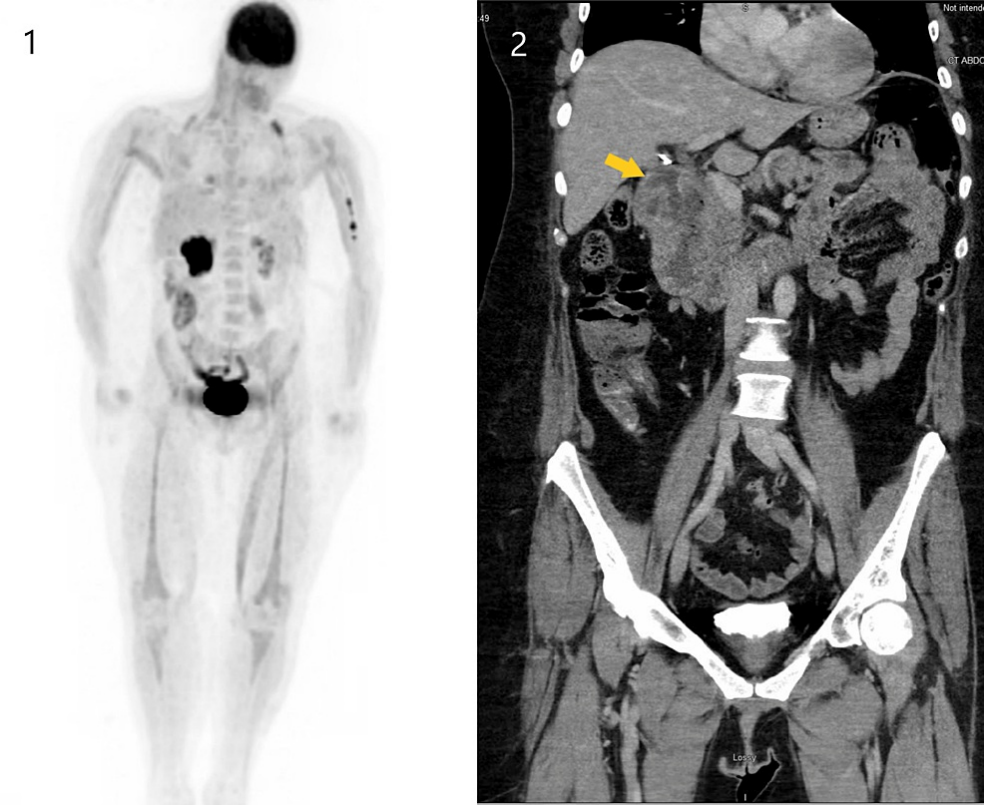
PET scan (1) and anteroposterior CT of the abdomen and pelvis (2) showing inflammation and increased uptake in the duodenum

**Figure 3:**
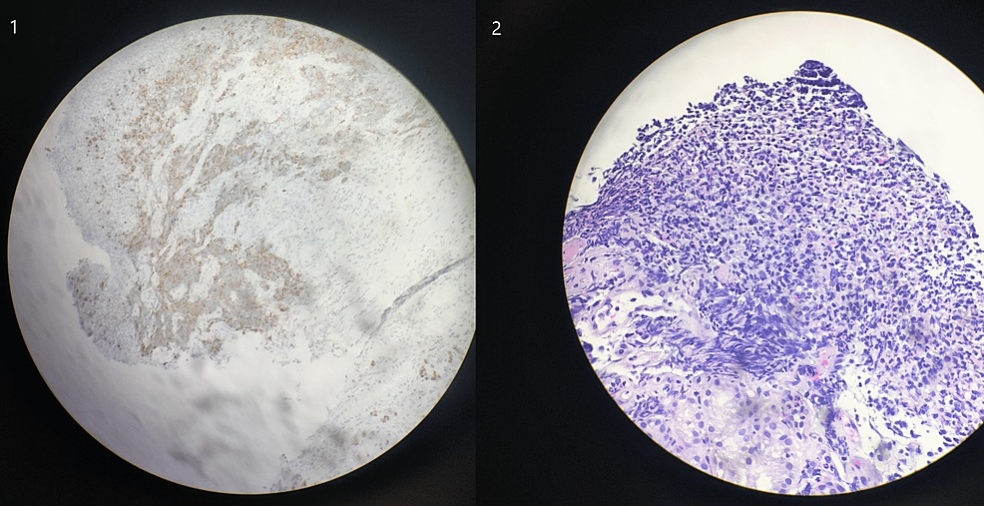
Synaptophysin immunostaining (1) and H&E immunostaining (2) Images courtesy of Michael Naski.

The patient's symptoms of generalized weakness and diplopia worsened substantially, and she eventually required inpatient hospital admission once again for expedited workup as well as empiric treatment. A positron emission tomography (PET) scan showed increased uptake in the duodenal region, which correlated with a thickening of the duodenum seen on a CT of the abdomen and pelvis earlier in her hospital course (Figure 2). Upper GI endoscopy showed a cratered duodenal ulceration near the duodenal bulb that was present on previous endoscopies. Biopsy showed poorly differentiated NEC, with positive synaptophysin, overexpression of p53, strong positivity with the Ki-67 proliferation marker in more than 90% of the neoplastic cells, and CDX2 suggesting a gastrointestinal origin (Figure 3). The findings were consistent with a limited-stage ESCC. Unfortunately, during the course of her inpatient workup, her respiratory status further declined, secondary to her generalized weakness, and she was intubated. Multiple attempts to wean the patient off ventilation were unsuccessful owing to her respiratory failure, which was deemed secondary to neuromuscular weakness. Later, a tracheostomy was performed due to ongoing ventilator dependence.

Intravenous immunoglobulin (IVIg), plasmapheresis, and rituximab were administered, with no appreciable symptom improvement. No apparent side effects were noted from the patient's treatments, as monitored by daily vitals and labwork. A Whipple procedure (also known as a pancreaticoduodenectomy) was recommended by surgical oncology; however, surgical risk related to recent rituximab and tenuous respiratory function barred operation. A consensus multidisciplinary decision was made in favor of treating the patient with carboplatin and etoposide in lieu of surgery.

**Figure 4:**
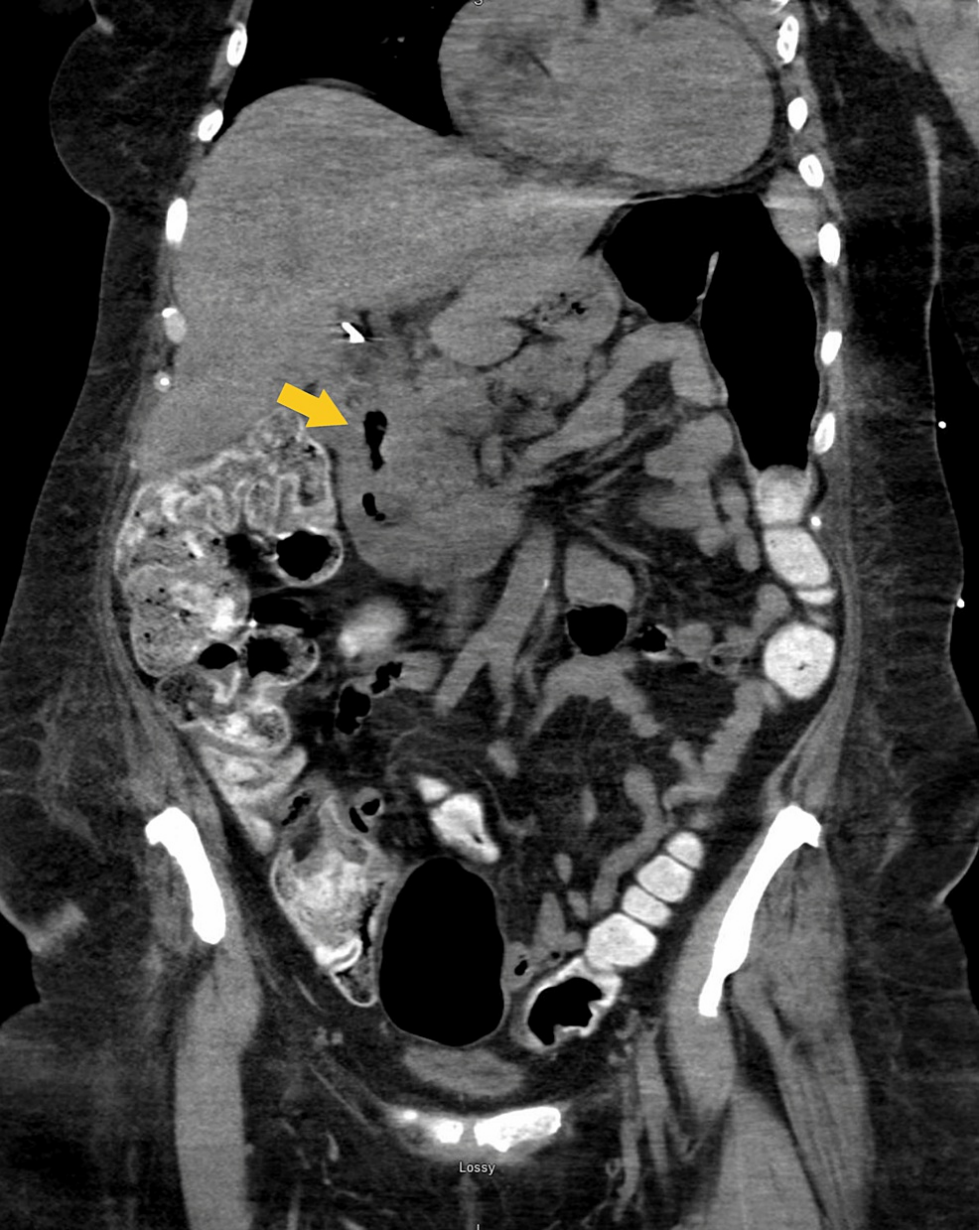
Anteroposterior CT of the abdomen and pelvis after five weeks of chemotherapy sessions

**Figure 5:**
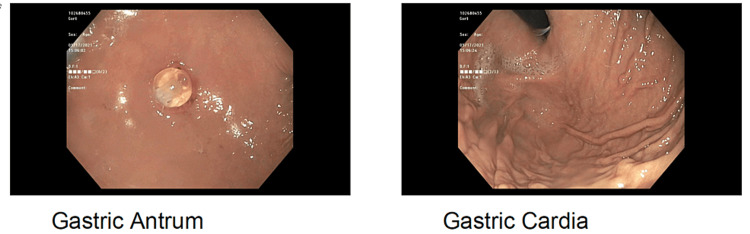
Images from post-chemotherapy endoscopy of gastric antrum and cardia Images courtesy of Kenneth Lown.

**Figure 6:**
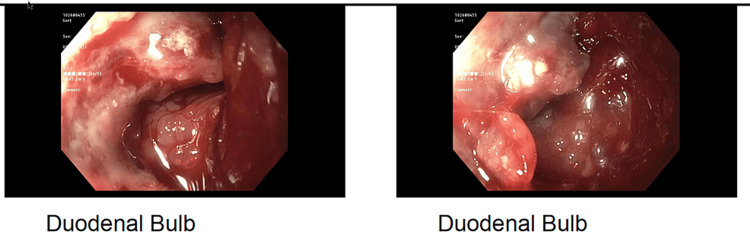
Further images from post-chemotherapy endoscopy, with re-demonstration of duodenal ulcerated mass Images courtesy of Kenneth Lown.

Despite the administration of chemotherapy over the next two months, repeat CT scans and endoscopy did not show improvement in the patient's ulceration (Figure 4). A continuing infiltrative mass in the duodenal bulb was appreciated on repeat endoscopy, with a noted deep circumferential ulceration with active oozing of blood (Figures 5-6). A repeat MRI of the brain later in the course did show improvement in the hyperintensity in the right lateral frontal lobe, thought to be a sign of a positive response to therapies (Figure 1). Unfortunately, no appreciable neurologic improvement was observed, and the patient remained ventilator-dependent. Further neurodiagnostic testing was also deferred due to clinical symptoms not improving despite treatment. The patient was deemed to have a low chance of meaningful recovery from an oncological standpoint. After extensive goals of care discussion, the patient ultimately decided to pursue comfort measures.

## Discussion

ESCC of the duodenum is a rare carcinoma for which there is sparse treatment data. The first documented case of an ESCC of the duodenum was by Swanson et al., upon postmortem evaluation of a patient who had metastatic carcinoma of the liver [8]. ESCCs of the duodenum are known to have similarities with their pulmonary counterparts, including an aggressive pattern and early metastasis [9]. Thus, the treatment of ESCCs parallels that of SCC of the lung with platinum-based chemotherapy [10]. Past cases have highlighted varying outcomes with treatment - a poorly-differentiated duodenal NET which also presented with paraneoplastic neurologic symptoms, had a partial response to platinum-based chemotherapy, and two cases of small bowel NET presenting as paraneoplastic myasthenia gravis responded to treatment with pyridostigmine and octreotide [11,12].

In terms of surgical treatments, resection or tumor excision has been studied more extensively in NETs, ranging from local excision to Whipple procedures [13,14]. The vast majority of NETs are non-functional tumors, and thus prognosis after treatment is generally favorable. There is comparatively fewer data on the resection of duodenal NECs (including ESCCs), likely due to more advanced disease on presentation [15,16]. Prognosis is generally worse than resection of NETs and dependent on the severity at onset as well as the staging of cancer. 

For this patient, the recommendations for treatment were unfortunately not based on widely available data and unfortunately did not result in beneficial outcomes in the treatment of symptoms or disease course. The treatments considered have shown benefits for adjacent diseases, however (especially SCC of the lung, and NETs as well as NECs), and thus there was a reasonable expectation of some treatment efficacy. This was ameliorated, however, by the timing of the treatments in the patient's disease course; immunotherapy and chemotherapy were administered after the patient had become debilitated and ventilator-dependent, and surgical resection was deferred due to too much operative risk, also owing to the patient's severe clinical status. 

## Conclusions

For our patient, the presentation of encephalomyelitis was a diagnostic challenge, with some noted limitations and strengths. Typically, most NETs and NECs will present only with GI symptoms, including melena, hematochezia, and increased gastric acid leading to duodenal ulceration. A limitation, in this case, was not initially considering a paraneoplastic process, which was influenced by an unremarkable initial biopsy. This was likely due to paraneoplastic syndromes appearing well before the malignancy was apparent. Furthermore, an early CT of the chest was not suggestive of SCC of the lung. There was also a limitation in the presence of established guidelines for treatment, as there was no initial consensus regarding the preferred treatment for this patient's ESCC of the small bowel. Ultimately, guidelines for adjacent diseases were referenced with recommendations for surgical resection per prior management of NETs and NECs of the small bowel, chemotherapy as per SCC of the lung, and immunotherapy as per previous paraneoplastic syndromes secondary to neuroendocrine tumors. The case was not without some strengths either, as the biopsy of the duodenal ulceration was strongly indicative of an ESCC, aiding in diagnostic certainty. This served as an anchor in the patient's later disease course, allowing treatment teams to move forward with their recommendations for treatment. Lastly, there was a strong interdisciplinary approach to the patient's care in terms of both diagnosis and treatment. Provider teams from specialties involved in the patient's care did recognize the rarity of the disease, the lack of established guidelines, and the severity of the patient's course, and collaborated extensively to still deliver the best standard of care.

The patient's clinical course spanned about a year, initially presenting with mild paresthesia and hematemesis, ending with ventilator dependence and severe generalized weakness. A multitude of treatments was all, unfortunately, without efficacy. Resection was not attempted, given the surgical risk and uncertainties of metastasis and neurologic recovery. ESCC of the small bowel is a rare disease and should be considered whenever paraneoplastic encephalomyelitis is suspected. Additionally, more research is needed into specific treatments for ESCC. This patient endured a period of diagnostic uncertainty and severe clinical symptoms, for which there may have been a different clinical course if ESCC had been deemed a prominent differential early on. For future patients with this illness, a different, perhaps more beneficial outcome is easier to imagine with earlier detection and better guidelines for treatment.

## References

[1] Ropper AH, Samuels MA, Klein JP, Prasad S (2019). Intracranial neoplasms and paraneoplastic disorders. Adams and Victor's Principles of Neurology.

[2] Seidenfeld J, Bonnell C, Ziegler KM (2006). Management of Small Cell Lung Cancer. https://www.ncbi.nlm.nih.gov/books/NBK38163/.

[3] Aamir MA, Sahebally SM, Gyorffy H, Aremu M (2017). Extra-pulmonary primary small-cell neuroendocrine carcinoma arising from the duodenum: rare tumor, unusual location. Clin Case Rep.

[4] Nagtegaal ID, Odze RD, Klimstra D (2020). The 2019 WHO classification of tumours of the digestive system. Histopathology.

[5] Fitzgerald TL, Dennis SO, Kachare SD, Vohra NA, Zervos EE (2015). Increasing incidence of duodenal neuroendocrine tumors: Incidental discovery of indolent disease?. Surgery.

[6] Adam VN, Budinčević H, Mršić V, Stojčić EG, Matolić M, Markić A (2013). Paraneoplastic limbic encephalitis in a patient with adenocarcinoma of the colon: a case report. J Clin Anesth.

[7] Sio TT, Paredes M, Uzair C (2012). Neurological manifestation of colonic adenocarcinoma. Rare Tumors.

[8] Swanson PE, Dykoski D, Wick MR, Snover DC (1986). Primary duodenal small-cell neuroendocrine carcinoma with production of vasoactive intestinal polypeptide. Arch Pathol Lab Med.

[9] Gonzalez RS (2020). Diagnosis and management of gastrointestinal neuroendocrine neoplasms. Surg Pathol Clin.

[10] Chandrasekharan C (2020). Medical management of gastroenteropancreatic neuroendocrine tumors. Surg Oncol Clin N Am.

[11] Nappi L, Formisano L, Damiano V, Matano E, Bianco R, Tortora G (2010). Paraneoplastic sensitive neuropathy associated with anti-hu antibodies in a neuroendocrine tumor of duodenum: a case report. Int J Immunopathol Pharmacol.

[12] Hermans MA, Stelten BM, Haak HR, de Herder WW, Dercksen MW (2014). Two patients with a neuroendocrine tumour of the small intestine and paraneoplastic myasthenia gravis. Endocrinol Diabetes Metab Case Rep.

[13] Vashistha N, Singhal D (2022). Current oncologic standards for surgery of small bowel cancers. J Gastrointest Cancer.

[14] Iwasaki T, Nara S, Kishi Y, Esaki M, Shimada K, Hiraoka N (2017). Surgical treatment of neuroendocrine tumors in the second portion of the duodenum: a single center experience and systematic review of the literature. Langenbecks Arch Surg.

[15] Dewan P, Bhat SP, Kishan Prasad HL, Ballal R, Sajitha K (2019). Neuroendocrine carcinoma of duodenum-an uncommon tumour at an unusual site. Indian J Surg Oncol.

[16] Watanabe M, Fujisaki S, Takashina M (2018). Two cases of neuroendocrine carcinoma of the non-ampullary duodenum (in Japanese). Cancer Chemot.

